# Supplementary material for: Transmembrane Protein TMEM230, Regulator of Glial Cell Vascular Mimicry and Endothelial Cell Angiogenesis in High-Grade Heterogeneous Infiltrating Gliomas and Glioblastoma
Source: Int J Mol Sci. 2024 Apr 3;25(7):3967. doi: 10.3390/ijms25073967 (PMC11011566; doi:10.3390/ijms25073967)
Supplement: Supplementary file 1 [file ijms-25-03967-s001.zip › ijms-2886023-supplementary.pdf]

Supplementary Table S2. Genes and pathways identified with increased glioma grade and TMEM230 upregulation in oligodendroglioma (ODG). TMEM230 regulates components of endomembrane system associated with cargo trafficking and secretion: Golgi complex and endoplasmic reticulum (see figure 7). Expression analysis correlated all genes differentially expressed with up-regulation of TMEM230 (p adjusted value of < or = 0.05, Supplementary Table 1). Gene Ontology and biological pathways were assessed using the False Discovery Rate method (p-adjusted value) as shown.

**Endomembrane system genes modulated in ODG with TMEM230-high vs TMEM230-low**	
**Glycosylation site: N-linked (p-adjusted value 5.77784E-34)**	
**Genes Down-regulated**	**Genes Up-regulated**
XYLT1, GPR173, CREB3L4, CREB3L1, GPR161, CHST11, GALR1, FLRT1, PCDHA4, LRRC37A2, GPR17, BTBD17, GRIN3A, PCDHB2, GPR27, MAMDC4, AHSG, TMC3, MMP16, ANGPTL2, RGR, DSCAML1, GPR45, COL11A2, ADAM22, SEZ6, GLRA1, ADAM29, GCNT2, CSMD3, SLC17A8, GPR158, BCHE, SULF2, SLC8A3, SMOC1, SEMA5A, ITGA2B, TNR, SLC38A1, KCNH7, SLC9A3, LPAL2, CLEC4F, COL17A1, SLC26A1, SLC14A2, LRIT2, CSPG5, ALK, TSPAN11, AMH, ADAMTS6, GRID2, SEZ6L, NLGN3, SLC24A3, LRP4, PTPRZ1, CACNG2, CACNG4, P2RX3, MDGA2, KCNK3, THSD7A, SLC6A18, KCNK10, STC2, SCN3A, PCDH20, LRRN4, LRRN1, IGSF9B, GRIK2, ROBO1, KCNT2, NOG, MST1, GFRA1, RGMB, GABBR1, GALNT13, CELSR3, DSCAM, CACNA1A, WSCD1, INHBC, BMP2, MEGF11, LRRTM3, LRRTM4, MADCAM1, ABCC8, PCDHGA3, SSTR2, CDHR1, LCAT, DLL1, ACAN, SLIT1, PCDHGC4, PCDHGC3, FAM155A	VIT, TMEM200B, COL12A1, MRGPRF, SLC4A2, SPINT2, F11R, GLDN, FGFRL1, ISLR, LIPC, GPR171, C3AR1, BVES, IER3, SERPINF1, SERPINF2, RNASE6, RNASE3, RNASE2, RNASE1, DKK2, SIGIRR, SPINT1, GPR160, PLBD1, SERPING1, CFB, CFH, HPN, CFI, CLCNKB, FPR1, IL20RA, PDGFA, LYPD3, FPR2, LYPD6, C2, ADAMTS16, C3, ADAMTS14, C6, C7, PDGFD, SERPINH1, PROM1, HLA-DQA2, HLA-DQA1, C19ORF38, ST3GAL3, NGFR, HLA-DRB5, FZD2, FZD5, ABCA5, MCAM, FUCA1, ABCA4, FZD6, FUCA2, FZD9, FZD8, ABCA7, CP, COL1A1, COL1A2, TNFSF8, HLA-DRB1, RET, SERPINA3, SERPINA1, SIGLEC9, WFIKKN2, PRSS23, PHEX, ASGR2, SERPINA5, MRC2, GPR132, ADAMTSL4, ADAMTSL3, TCTN2, MRC1, CTLA4, OLR1, EMB, TIMP1, CTBS, HLA-DPA1, HAVCR2, CHST8, CHST6, CLUL1, GPR37, SLC2A10, NFAM1, MAMDC2, TMC8, EMP1, EMP3, F3, BACE2, CD200R1, COL2A1, GPRC5A, SLC7A7, ANGPTL7, COL21A1, GAS6, CHST2, HLA-DQB1, CRB2, GCNT1, PTAFR, FSTL1, PTGS1, HLA-DMA, ADAM28, HLA-DMB, FRZB, OLFML3, TMEM67, OLFML1, APOL1, IL12RB1, C11ORF45, RNF130, LEFTY2, LRRC25, EVC2, P2RY13, IL10RA, GPR55, ADAM33, GPR1, VWA3A, PRSS36, KIAA2013, SULF1, PLXDC2, TMEM156, GPR143, SLCO3A1, RNF149, CLCF1, HLA-DPB1, PLP2, FMOD, LGR6, CCL28, OLFML2B, CSF3R, GPR65, TNC, F13A1, PLOD1, EFEMP2, HPGDS, PODNL1, EFEMP1, ADORA3, LAMP3, CLEC5A, TNFRSF8, HLA-DOA, SLC39A4, IL13RA1, LAG3, TICAM2, COL27A1, BSCL2, MFNG, ROR1, FKBP9, ROR2, DSC2, CHODL, CSF1R, CD274, KCNE1, KCNE3, GPR88, TACSTD2, LY96, TNFRSF11B, AEBP1, GPR84, TNFRSF11A, SLC9A1, CLEC7A, TNFRSF14, SLC15A3, CD300LF, NPTX2, PLTP, CPA4, KL, CD163, SLC37A2, HTR1D, LY86, CYBRD1, LRRC66, IGSF10, PTPRC, P4HA3, ITGA11, OGN, HLA-DRA, SP9, CNTNAP3, KCNK5, MOXD1, ST6GALNAC2, ITGAM, ITGB5, COL14A1, ITGB4, ITGB3, ITGB2, LECT1, ECE1, TREM2, CPZ, ITGAL, MSLN, WISP1, TSPAN10, ADAMTS4, SPN, SCPEP1, FCGR3A, ADAMTS3, EMILIN3, EMILIN2, ITGB7, EMILIN1, TNFSF12-TNFSF13, ADAMTS7, SLC13A3, RNASET2, ITGA2, ADRA2C, TNFRSF1B, ADRA2A, ASPN, TNFRSF1A, NPC2, DSG2, MET, ST6GALNAC5, COL15A1, SEMA3C, C1S, FRRS1, C1R, UNC93B1, SEMA3A, FGL2, NTN5, SEMA3F, IL2RG, COCH, CECR1, P2RY6, P2RY2, CEACAM21, FCGR1A, FCGR1B, CTHRC1, LINGO2, SEMA4D, ANGPT1, MBOAT7, GZMA, GZMH, MFAP5, MFAP4, FCGR2A, FRAS1, CLEC2B, TSPAN19, P2RX1, IL2RB, PI16, EVI2B, FCGR2B, FOLR2, FOLR1, CBLN3, FCGR2C, CD244, OXTR, PRF1, LDLRAD2, ICAM4, KLHDC7A, AQP5, IFI30, LCTL, AQP3, ICAM1, AQP1, C4A, NIPAL3, OXER1, EPHB2, SYNPO, ICOSLG, HABP2, SLC34A2, IL15RA, EPHA4, EPHA7, SLC6A16, IL1R1, SLC11A1, KCNK13, TIMD4, HLA-G, GBGT1, SFRP4, FNDC1, CD109, KCNQ1, CD226, CD101, CES1, SLC45A2, IGSF3, GYPC, IGSF1, EBI3, LPAR1, TMTC2, CD79B, CD79A, CD19, ST8SIA4, ARSI, SLAMF6, TSPAN2, CD14, APOB, SLAMF1, CCR1, NTNG1, MSR1, SIGLEC14, SIGLEC11, TNFSF13, SIGLEC10, GDF5, VASN, BST2, BST1, TG, LPAR5, ARTN, LPAR6, SDC1, PTPRU, COL18A1, CD40, SLC44A3, ECM2, CTSZ, SECTM1, CTSW, GRIK1, CD3D, WFDC2, CTSS, PTPRF, SLC22A15, STAB1, CTSH, CD37, CTSC, CD33, CCR2, IL10, CD53, CD52, IL15, ANO9, ANO6, MMEL1, PDCD1LG2, GFRA3, OSMR, GFRA2, TLR1, CEACAM1, B3GNT8, CDCP1, NOV, B3GNT7, B3GNT5, TLR8, VWA1, B3GNT2, TLR7, CD302, TLR5, CD44, MATN2, TLR2, PTGER4, C1QA, SELPLG, TNXB, TMPRSS3, PTGER2, PTGER3, AMIGO2, GDPD2, CXCR6, CST7, LILRA2, RELN, CXCR3, KISS1R, GPC3, SUSD3, GPC4, CD58, GABRE, CD55, LAIR1, MICB, MPZL2, CD74, CD72, GDF15, SUSD2, DKKL1, OSM, LILRB1, LILRB2, FAP, QPCT, CACHD1, F2RL1, CD69, CD68, F2RL2, CD86, FCGBP, STEAP3, CD83, HFE, SIRPG, NPY2R, MPL, CLEC18C, LOXL4, CLEC18B, PLAT, CELSR1, CLEC18A, PLB1, PLAU, SIT1, C1RL, FAM3B, ENPP1, PRND, CD96, POSTN, CD300A, APLP1, CYBB, PCOLCE, ATP1B1, PDGFRL, OSCAR, LOX, SCNN1A, ADAM12, COL4A6, COL4A5, CD300C, KLRB1, C5AR1, CHRNA9, FBLN1, CSF2RB, LTBP2, SLC1A5, CACNA1F, FBLN2, NID2, CSF2RA, EFNA4, LTBP1, PLD3, FBLN5, DPP4, EFNB2, CACNA1S, WNT2, WNT4, TGFB2, OPN3, VCAM1, TGFB1, PTCH2, RARRES1, CRTAM, TRPV2, EPHX3, PROC, COL5A2, FGFR3, ULBP1, ULBP3, WNT2B, B4GALT1, MEGF10, TNFAIP6, SIRPB2, SLC2A5, HS2ST1, BDKRB2, MAN1A1, CA9, SCN5A, ABCC3, EGFL6, PLAUR, WNT16, MMP9, TGFBR3, GREM1, RCN3, COL6A2, TMEM217, CHI3L2, CASQ2, CHI3L1, COL6A3, LTB, LTF, COLEC12, LAMA2, TM2D1, LAMA4, TFPI, THBS1, KDELC2, MAN1C1, SCN7A, LRIG3, SLIT3, S1PR3, CHSY3, TAS1R1, GALNT4, GALNT3, LGI4, EGF, LAMB1, SOD3, HS3ST1, CD2, CD4, CD6, CD5, CD7, PMP22, LTBR
**Endoplasmic reticulum Lumen (Benjamini padj 3.41952E-06)**	
**Genes Down-regulated**	**Genes Up-regulated**

COL17A1, COL11A2, BCHE	COL18A1, COL15A1, SERPINA1, COL14A1, COL12A1, CTSZ, PDGFA, THBS1, LIPC, KDELC2, ADAMTSL4, PDGFD, SERPINH1, ARSI, APOB, CTSC, WNT4, ADAMTS7, COL27A1, RNASET2, PLAUR, GPX8, GPX7, PDIA5, RCN3, COL1A1, COL3A1, CD4, COL2A1, PROC, COL1A2, COL5A1, P4HA3, COL6A2, COL5A2, COL4A6, COL8A2, COL4A5, RDH5, COL6A3, COL8A1, COL21A1, ERP27, GAS6, CES1

Endocytic vesicle membrane (p-adjusted value 4.8375E-04)

Genes Down-regulated	Genes Up-regulated
GPR161, CACNG2, CACNG4	COLEC12, MSR1, CD74, CD163, HLA-DRB5, STAB1, HLA-DPB1, AP2S1, HLA-DRA, PICK1, HLA-DQA2, HLA-DQA1, HLA-DQB2, HLA-DRB1, WLS, HLA-DPA1, HLA-DQB1, WNT4

Proteoglycans in cancer (p-adjusted value 7.62698E-04)

Genes Down-regulated	Genes Up-regulated
ANK1	WNT2B, ITGB5, SDC4, ITGB3, SDC2, PIK3CD, TWIST1, IQGAP1, THBS1, PIK3CG, SLC9A1, PIK3R5, CDC42, PLAU, PLCG2, GPC3, FLNA, PLCE1, FLNC, WNT2, WNT4, TGFB2, TGFB1, FZD2, FZD5, LUM, HGF, ITGA2, FZD6, FZD9, PLAUR, IGF2, FZD8, MSN, IGF1, WNT16, MMP9, MAPK13, SDC1, HCLS1, PTPN6, MET, CD44, TLR2

Proteoglycan (p-adjusted value 1.097553E-03)

Genes Down-regulated	Genes Up-regulated
ACAN, TNR, CSPG5	SRGN, CD74, SDC4, LUM, SDC2, TGFBR3, SRPX2, GPC3, SDC1, GPC4, FMOD, CD44, ADAMTS7

Supplementary Table S3. Genes and pathways identified with increased glioma grade and TMEM230 upregulation. TMEM230 regulates motor protein dependent intracellular trafficking and secretion of metalloproteinases (ADAMs and MMPs) and phagosomes for microchannel formation. (see figure 7). Expression analysis correlated all genes differentially expressed with up-regulation of TMEM230 (p adjusted value of < or = 0.05, Supplementary Table 1). Gene Ontology and biological pathways were assessed using the False Discovery Rate method as shown.

Trafficked and secreted factor and vesicle associated genes modulated in ODG with TMEM230-high vs TMEM230-low

Secreted (p-adjusted value 6.51983E-25)

Genes Down-regulated	Genes Up-regulated
XYLT1, THSD7A, CHGB, STC2, FLRT1, BTBD17, CRTAC1, FGFBP3, AHSG, NOG, MST1, **MMP16**, ANGPTL2, DNASE1L3, COL11A2, BCHE, GABBR1, SMOC1, TNR, DSCAM, LPAL2, COL17A1, PDZD2, INHBC, BMP2, VWC2L, AMH, **ADAMTS6**, LCAT, ACAN, PTPRZ1, SLIT1, TUB	VIT, SPON2, COL12A1, PRF1, ICAM4, IFI30, GLDN, C4A, LGALS3, ISLR, LGALS1, LIPC, PROK2, LGALS9, HABP2, FAM19A3, IL15RA, MIA, IL1R1, SERPINF1, SERPINF2, RNASE6, RNASE4, RNASE3, RNASE1, DKK2, SFRP4, SPINT1, FNDC1, COL8A2, SERPING1, COL8A1, NBL1, CFB, CFH, SDC4, DHH, IGSF1, CFI, EBI3, PDGFA, PLA2G5, LYPD6, C2, **ADAMTS16,** C3, **ADAMTS14,** C6, C7, SCGB1D2, PDGFD, S100A13, ARSI, CD14, APOB, ST3GAL3, SLAMF1, GSDMD, TNFSF13, SIGLEC10, FUCA2, IGF2, IGF1, PGA3, GDF5, CP, PGA5, VASN, PGA4, COL1A1, COL1A2, TG, ARTN, SDC1, HAMP, SERPINA3, COL18A1, CD40, SERPINA1, ECM2, C2CD2, WFIKKN2, SECTM1, PRSS23, WFDC2, SERPINA5, ESM1, **ADAMTSL4,** GNLY, **ADAMTSL3,** OLR1, TIMP1, IL10, CLUL1, ANXA1, FGFBP2, ANXA2, IL15, MAMDC2, IL18, MMEL1, PDCD1LG2, OTOS, F3, CEACAM1, CD200R1, COL2A1, CDCP1, NOV, ANGPTL7, COL21A1, VWA1, GAS6, MATN2, C1QB, CRB2, C1QA, CTF1, TNXB, LRRC17, CAPG, LILRA2, CST7, FSTL1, APOL4, **ADAM28,** RELN, SRPX2, OLFML3, FRZB, OLFML1, GPC3, GPC4, C11ORF45, APOL1, LEFTY2, CD55, CCL25, CCL22, GSN, RNLS, CCL20, GDF15, OSM, DKKL1, LILRB1, VWA3A, PRSS36, COL3A1, FAP, ADM2, QPCT, CLCF1, C16ORF89, CCDC3, FMOD, CCL28, C1QC, FCGBP, OLFML2B, CSF3R, TNC, CLEC18C, CLEC18B, F13A1, LOXL4, PLAT, FGF1, CXCL14, CLEC18A, LOXL1, EFEMP2, PODNL1, EFEMP1, PLAU, C1RL, FAM3B, ENPP1, POSTN, COL27A1, IGFBP2, PCOLCE, NPNT, THNSL2, PDGFRL, OSCAR, LOX, CD8A, **ADAM12,** COL4A6, COL4A5, LY96, FBLN1, C1ORF54, TNFRSF11B, LTBP2, AEBP1, NTS, FBLN2, NID2, EFNA4, CSF2RA, LTBP1, FBLN5, DPP4, NLRP3, CYTL1, IGFBP6, NPTX2, PLTP, WNT2, CPA4, WNT4, KL, CD163, TGFB2, TGFB1, RARRES2, LY86, EPHX3, ISG15, ABHD15, CXCL10, IGSF10, CXCL11, CXCL12, PROC, COL5A1, COL5A2, OGN, ANXA2P2, FGFR3, CNTNAP3, CXCL6, CXCL9, B4GALT1, WNT2B, C2ORF40, COL14A1, LECT1, TREM2, TRH, CPZ, DMKN, MSLN, WISP1, CYR61, **ADAMTS4,** SCPEP1, FCGR3A, **ADAMTS3**, CASP4, EMILIN3, EMILIN2, EMILIN1, TNFSF12-TNFSF13, C3ORF58, **ADAMTS7**, SRGN, **MMP7**, EGFL6, RNASET2, PLAUR, GPX7, WNT16, C1QL4, TNFRSF1B, **MMP9**, ASPN, TNFRSF1A, TGFBR3, GREM1, OAS1, NPC2, COL6A2, CHI3L2, CHI3L1, COL6A3, ANG, MET, LTF, COLEC11, COL15A1, SEMA3C, LAMA2, SEMA3A, LAMA4, FGL2, NTN5, SEMA3F, TFPI, PTHLH, COCH, CECR1, CCL5, SLIT3, GBP1, CTHRC1, ANGPT1,

	LGI4, LUM, GZMA, LAMB1, SOD3, MFAP5, GZMK, MFAP4, CD6, C9ORF47, APOC2, MFAP2, HCG22, PI16, FOLR2, CBLN3, FOLR1
**Phagosome (p-adjusted value 2.61672E-07)**	
**Genes Down-regulated**	**Genes Up-regulated**
	COLEC12, COLEC11, ITGAM, ITGB5, NCF1, C1R, NCF2, ITGB3, NCF4, ITGB2, TCIRG1, CORO1A, THBS1, CTSS, C3, MRC2, TUBA1C, TUBA1B, TUBB6, HLA-DMA, FCGR3A, HLA-DMB, CLEC7A, MRC1, OLR1, CD14, FCGR1A, HLA-DOA, HLA-DQA2, HLA-DQA1, HLA-DPA1, MSR1, ATP6V0B, HLA-DRB5, ITGA2, CYBA, HLA-G, FCGR2A, HLA-DPB1, HLA-DRA, FCGR2B, FCGR2C, HLA-DRB1, HLA-DQB1, TLR2, VAMP3
**Motor protein (p-adjusted value 5.3829E-04)**	
**Genes Down-regulated**	**Genes Up-regulated**
KIF13A, KIF21B, MYO7B, MYO3B, KIF26B, MYH6, MYH7	ACAA2, DNAH5, DNAH6, DNAH9, KIF17, MYL12A, DNALI1, MYH11, DNAH11, DYNLT3, KIF23, MYO7A, MYO1D, CENPE, KIF18A, KIF16B, MYO5B, MYO3A, MYO5C, DYNLRB2, KIF2C, MYL9, MYO1F, MYO1G

Supplementary Table S4. Genes and pathways identified with increased glioma grade and TMEM230 upregulation. TMEM230 promoted Golgi complex dependent upregulation of glycoproteins and angiogenesis in highly vascularized infiltrating gliomas. Expression analysis correlated all genes differentially expressed with up-regulation of TMEM230 (p adjusted value of < or = 0.05, Supplementary Table 1). Gene Ontology and biological pathways were assessed using the False Discovery Rate method as shown.

**Glycoproteins and angiogenesis associated genes modulated in ODG with TMEM230-high vs TMEM230-low**	
**Glycoprotein (p-adjusted value 5.5377E-43)**	
**Genes Down-regulated**	**Genes Up-regulated**
XYLT1, GPR173, CREB3L4, CREB3L1, CHGB, FIBCD1, GPR161, CHST11, GALR1, FLRT1, PCDHA4, LRRC37A2, GPR17, BTBD17, GRIN3A, PCDHB2, GPR27, MAMDC4, AHSG, TET1, TMC3, MMP16, ANGPTL2, RGR, DSCAML1, GPR45, COL11A2, ADAM22, SEZ6, GLRA1, ADAM29, GCNT2, CSMD3, SLC17A8, GPR158, ZNF462, BCHE, SULF2, SLC8A3, SMOC1, SEMA5A, ITGA2B, TNR, SLC38A1, KCNH7, SLC9A3, LPAL2, CLEC4F, COL17A1, SLC26A1, SLC14A2, LRIT2, CSPG5, ALK, TSPAN11, AMH, ADAMTS6, GRID2, TRPC3, SEZ6L, NLGN3, SLC24A3, LRP4, PTPRZ1, CACNG2, CACNG4, P2RX3, MDGA2, KCNK3, THSD7A, SLC6A18, KCNK10, STC2, SCN3A, PCDH20, LRRN4, LRRN1, IGSF9B, GRIK2, ROBO1, KCNT2, CRTAC1, NOG, MST1, GFRA1, RGMB, GABBR1, GALNT13, CELSR3, DSCAM, SHISA9, CACNA1A, WSCD1, INHBC, BMP2, MEGF11, LRRTM3, LRRTM4, MADCAM1, ABCC8, PCDHGA3, SSTR2, CDHR1, RGS17, LCAT, DLL1, ACAN, SLIT1, PCDHGC4, PCDHGC3, FAM155A	VIT, TMEM200B, COL12A1, MRGPRF, SLC4A2, SPINT2, F11R, GLDN, FGFRL1, ISLR, LIPC, GPR171, C3AR1, BVES, IER3, SERPINF1, SERPINF2, RNASE6, RNASE3, RNASE2, RNASE1, DKK2, SIGIRR, SPINT1, GPR160, PLBD1, SERPING1, CFB, CFH, HPN, CFI, CLCNKB, FPR1, IL20RA, PDGFA, LYPD3, FPR2, LYPD6, C2, ADAMTS16, C3, ADAMTS14, C6, C7, PDGFD, SERPINH1, PROM1, HLA-DQA2, HLA-DQA1, C19ORF38, ST3GAL3, NGFR, HLA-DRB5, FZD2, JUP, FZD5, ABCA5, MCAM, FUCA1, ABCA4, FZD6, FUCA2, FZD9, FZD8, ABCA7, CP, COL1A1, COL1A2, SNAI1, TNFSF8, FXYD5, HLA-DRB1, RET, SERPINA3, SERPINA1, SIGLEC9, WFIKKN2, VSIG10L, PRSS23, PHEX, ASGR2, SERPINA5, MRC2, GPR132, ADAMTSL4, ADAMTSL3, TCTN2, MRC1, CTLA4, OLR1, EMB, TIMP1, CTBS, HLA-DPA1, HAVCR2, CHST8, CHST6, CLUL1, GPR37, SLC2A10, NFAM1, MAMDC2, TMC8, EMP1, EMP3, F3, BACE2, CD200R1, COL2A1, GPRC5A, SLC7A7, ANGPTL7, TMEM106C, COL21A1, GAS6, HCST, HLA-DQB2, CHST2, HLA-DQB1, CRB2, GCNT1, PTAFR, FSTL1, PTGS1, HLA-DMA, ADAM28, HLA-DMB, SRPX2, FRZB, OLFML3, TMEM67, OLFML1, APOL1, IL12RB1, C11ORF45, RNF130, LEFTY2, LRRC25, EVC2, P2RY13, IL10RA, GPR55, ADAM33, GPR1, VWA3A, PRSS36, KIAA2013, SULF1, PLXDC2, TMEM156, COL3A1, GPR143, SLCO3A1, RNF149, CLCF1, HLA-DPB1, C16ORF89, PLP2, CCDC3, FMOD, LGR6, CCL28, OLFML2B, CSF3R, GPR65, TNC, F13A1, PLOD1, EFEMP2, PODNL1, EFEMP1, ADORA3, LAMP3, CLEC5A, TNFRSF8, HLA-DOA, SLC39A4, IL13RA1, LAG3, COL27A1, IGFBP2, BSCL2, MFNG, ROR1, FKBP9, ROR2, DSC2, CHODL, CSF1R, CD274, KCNE1, KCNE3, GPR88, TACSTD2, LY96, TNFRSF11B, AEBP1, GPR84, TNFRSF11A, SLC9A1, CLEC7A, TNFRSF14, IGFBP6, SLC15A3, CD300LF, NPTX2, PLTP, CPA4, KL, CD163, SLC37A2, HTR1D, LY86, CYBRD1, LRRC66, IGSF10, PTPRC, P4HA3, ITGA11, OGN, HLA-DRA, CNTNAP3, KCNK5, MOXD1, ST6GALNAC2, ITGAM, ITGB5, COL14A1, ITGB4, ITGB3, ITGB2, LECT1, ECE1, TREM2, CPZ, ITGAL, MSLN, WISP1, TSPAN10, ADAMTS4, SPN, SCPEP1, FCGR3A, ADAMTS3, EMILIN3, EMILIN2, ITGB7, EMILIN1, TNFSF12-TNFSF13, ADAMTS7, SLC13A3, RNASET2, ITGA2, ADRA2C, TNFRSF1B, ADRA2A, ASPN, TNFRSF1A, NPC2, DSG2, MET, ST6GALNAC5, CEBPB, COL15A1, SEMA3C, C1S, FRRS1, C1R, UNC93B1, SEMA3A, FGL2, NTN5, SEMA3F, IL2RG, COCH, CECR1, CDC42, P2RY6, CCL5, P2RY2, CEACAM21, FCGR1A, FCGR1B, KCNJ1, CTHRC1, LINGO2, SEMA4D, ANGPT1, MBOAT7, GZMA, GZMH, MFAP5, MFAP4, FCGR2A, FRAS1, CLEC2B, TSPAN19, APOC2, P2RX1, MFAP2, IL2RB, HCG22, CD248, PI16, EVI2B, FCGR2B, FOLR2, FOLR1, CBLN3, FCGR2C, CD244, SPON2, OXTR, PRF1, LDLRAD2, ICAM4, KLHDC7A, AQP5, IFI30, LCTL, AQP3, ICAM1, AQP1, C4A, NIPAL3, OXER1, EPHB2, SYNPO, ICOSLG, HABP2, SLC34A2, IL15RA, EPHA4, EPHA7, ACSL1, SLC6A16, IL1R1, SLC11A1, KCNK13, KRT8, TIMD4, HLA-G, GBGT1, SFRP4, FNDC1, CD109, KCNQ1, CD226, CD101, CES1, SLC45A2, SDC4, IGSF3, GYPC, SDC2, IGSF1, GYPE, EBI3, LPAR1, TMTC2, CD79B, CD79A, CD19, ST8SIA4, ARSI, SLAMF6, TSPAN2, TRPM8, CD14, APOB, SLAMF1, CCR1, NTNG1, SIGLEC16, MSR1, SIGLEC14, SIGLEC11, TNFSF13, SIGLEC10, IGF2, GDF5, VASN,

	BST2, BST1, TG, LPAR5, ARTN, LPAR6, SDC1, PTPRU, COL18A1, CD40, SLC44A3, ECM2, CTSZ, SECTM1, CTSW, GRIK1, CD3D, WFDC2, CTSS, PTPRF, UCHL1, ESM1, SLC22A15, STAB1, RAC2, CTSH, CD37, CCR5, CTSC, CD33, CCR2, IL10, CD53, CD52, IL15, ANO9, ANO6, MMEL1, PDCD1LG2, HBA1, RHOC, GFRA3, OSMR, GFRA2, TLR1, CEACAM1, B3GNT8, CDCP1, NOV, B3GNT7, B3GNT5, TLR8, VWA1, B3GNT2, RDH5, TLR7, CD302, TLR5, CD44, MATN2, TLR2, PTGER4, C1QB, C1QA, SELPLG, TNXB, TMPRSS3, PTGER2, PTGER3, AMIGO2, GDPD2, CXCR6, CST7, LILRA2, RELN, CXCR3, PDPN, KISS1R, GPC3, SUSD3, GPC4, CD58, GABRE, CD55, LAIR1, MICB, MPZL2, CD74, CD72, GDF15, SUSD2, DKKL1, OSM, LILRB1, LILRB2, XG, KRT18, FAP, QPCT, CACHD1, F2RL1, CD69, CD68, F2RL2, C1QC, CD86, FCGBP, STEAP3, CD83, HFE, SIRPG, NPY2R, MPL, CLEC18C, LOXL4, CLEC18B, PLAT, CELSR1, CLEC18A, PLB1, PLAU, SIT1, C1RL, FAM3B, ENPP1, PRND, CD96, POSTN, CD300A, HGF, APLP1, CYBB, PCOLCE, ATP1B1, PDGFRL, OSCAR, LOX, SCNN1A, ADAM12, COL4A6, COL4A5, CD300C, KLRB1, C5AR1, CHRNA9, FBLN1, CSF2RB, LTBP2, SLC1A5, CACNA1F, FBLN2, NID2, CSF2RA, EFNA4, LTBP1, PLD3, FBLN5, DPP4, EFNB2, CACNA1S, WNT2, WNT4, C16ORF54, TGFB2, OPN3, VCAM1, TGFB1, PTCH2, CRTAM, TRPV2, CLEC17A, EPHX3, KIF18A, PROC, COL5A2, TMEM119, FGFR3, ULBP1, ULBP3, WNT2B, B4GALT1, MEGF10, TNFAIP6, SIRPB2, SLC2A5, DMKN, HS2ST1, BDKRB2, MAN1A1, CA9, SCN5A, SRGN, ABCC3, EGFL6, PLAUR, WNT16, MMP9, TGFBR3, GREM1, RCN3, OAS2, COL6A2, TMEM217, CHI3L2, CASQ2, CHI3L1, COL6A3, LTB, LTF, COLEC12, HIST1H2BJ, LAMA2, TM2D1, LAMA4, HIST1H2BK, TFPI, THBS1, KDELC2, MAN1C1, SCN7A, LRIG3, SLIT3, S1PR3, CHSY3, TAS1R1, GALNT4, GALNT3, LGI4, EGF, LUM, LAMB1, SOD3, HS3ST1, CD2, CD4, CD6, CD5, CD7, PMP22, VIM, LTBR
**Angiogenesis associated genes (p-adjusted value 5.273066E-03)**	
**Genes Down-regulated**	**Genes UP-regulated**
HEY1, THSD7A	COL18A1, COL15A1, SH2D2A, PDGFA, FGF1, HOXB13, MEOX2, PIK3CG, TYMP, ESM1, SRPX2, GJA5, CXCR3, HOXA3, PROK2, CYP1B1, PTK2B, HMOX1, TNFSF12-TNFSF13, EPHB2, HOXA7, HTATIP2, VAV3, TGFB2, JUN, TNFRSF12A, SYK, ANGPT1, ANXA2, FZD5, EGF, MCAM, IL18, FZD8, MMP14, CEACAM1, KLF5, FAP, NOV, ADM2, COL8A2, HOXB3, COL8A1, ANG

Supplementary Table S5. Most significant glycoproteins and angiogenesis associated genes in ODG with TMEM230 upregulation. Genes were selected based on an adjusted P-value (< 1,00E-05) and an absolute log2 fold change of > 2.

**OLIGODENDROGLIOMA**		
**Glycoproteins (p-adjusted value 5.54E-43)**		
**Down-regulated in TMEM230 high vs TMEM230 low**		
**Gene name**	**log2FoldChange>2**	**padj<1,00E-05**
TMC3	-2,503399205 [1]	4,71168E-08 [2]
**Up-regulated in TMEM230 high vs TMEM230 low**		
**Gene name**	**log2FoldChange>2**	**padj<1,00E-05**
ABCC3	2,426369431	1,53651E-15
ANO9	2,136173506	4,68867E-05
CD2	2,118803466	2,18233E-12
CD3D	2,479285615	8,23728E-08
CELSR1	2,258563899	7,53705E-21
COL2A1	2,085797221	1,12395E-06
CXCR6	2,106246226	1,29299E-09
C6	3,460983598	4,75713E-09
CHI3L1	4,259222655	1,60513E-39
CHI3L2	2,16443761	7,55398E-15
EMILIN3	2,552719902	2,2874E-13
EVC2	2,190079011	4,52538E-17
GPR1	2,324815684	2,88324E-05
GPRC5A	2,030586314	1,65161E-12
GZMA	2,113054151	6,88925E-11
IGFBP2	2,313066319	2,7526E-17
KLRB1	2,011507458	1,37078E-06
KISS1R	2,130302848	1,13059E-05
LECT1	2,073242774	3,52722E-10
MMP9	2,373232832	5,48088E-10
MMEL1	3,599237766	1,87916E-14
MOXD1	3,25804939	1,3944E-23
POSTN	2,832912181	1,06319E-11
ROR2	2,225722267	3,63308E-18
SIT1	2,528683236	4,56323E-09

SLC34A2	3,015907839	3,09943E-13
TSPAN19	2,518618103	1,78963E-06
ULBP1	2,213250722	3,9782E-11
XG	2,206443316	2,10978E-05
**Angiogenesis associated genes (p-adjusted value 5.273E-03)**		
**Down-regulated in TMEM230 high vs TMEM230 low**		
**Gene name**	**log2FoldChange>2**	**padj<1,00E-05**
TMC3	-2,503399205	4,71168E-08
**Up-regulated in TMEM230 high vs TMEM230 low**		
**Gene name**	**log2FoldChange>2**	**padj<1,00E-05**
HOXA3	4,342084814	4,16713E-05
HOXA7	7,695907211	1,02553E-22
HOXB3	4,284650669	2,64264E-24
HOXB13	3,743738029	4,55944E-06
CXCR3	2,54484793	4,18208E-08
COL8A1	2,465517007	4,97546E-16

Supplementary Table S6. Comparison of Fold change and p adjusted (padj) value in expression of glycoproteins and angiogenesis associated genes in ODG or GBM with TMEM230 upregulation. Selected genes (Supplementary Table 1) identified in ODG (with an adjusted P-value (< 1.00E-05) and an absolute log2 fold change of > 2) were compared to genes in patients of GBM.

**ODG**				**GBM**		
**Glycoproteins**						
**Down-regulated in TMEM230 high vs TMEM230 low**						
**Gene name**	**log2FC**	**padj**		**Gene name**	**log2FC**	**padj**
TMC3	- 2,503399205	4,71168E-08		TMC3	0.53180330413885	0.062056137752
**Up-regulated in TMEM230 high vs TMEM230 low**						
**Gene name**	**log2FC**	**padj**		**Gene name**	**log2FC**	**padj**
ABCC3	2,426369431	1,53651E-15		ABCC3	0.0212250366	0.95055705
ANO9	2,136173506	4,68867E-05		ANO9	0.0689326870	0.90078775
CD2	2,118803466	2,18233E-12		CD2	0.6315172698	0.01887520
CD3D	2,479285615	8,23728E-08		CD3D	0.9245315466	0.00235036
CELSR1	2,258563899	7,53705E-21		CELSR1	0.3540866600	0.22458230
COL2A1	2,085797221	1,12395E-06		COL2A1	1.1396620861	0.00298383
CXCR6	2,106246226	1,29299E-09		CXCR6	0.5807445409	0.050250776
C6	3,460983598	4,75713E-09		C6	0.8509950950	0.22063700
CHI3L1	4,259222655	1,60513E-39		CHI3L1	0.8561575969	0.02064791
CHI3L2	2,16443761	7,55398E-15		CHI3L2	1.05442912647	0.000361115
EMILIN3	2,552719902	2,2874E-13		EMILIN3	0.2712692642	0.403328296
EVC2	2,190079011	4,52538E-17		EVC2	0.2283639486	0.333497337
GPR1	2,324815684	2,88324E-05		GPR1	0.3253671064	0.536369244
GPRC5A	2,030586314	1,65161E-12		GPRC5A	0.2743910284	0.325367106
GZMA	2,113054151	6,88925E-11		GZMA	1.0158684935	0.000248458
IGFBP2	2,313066319	2,7526E-17		IGFBP2	0.2835995101	0.178020221
KLRB1	2,011507458	1,37078E-06		KLRB1	1.0200424914	0,002878009
KISS1R	2,130302848	1,13059E-05		KISS1R	0.9653430216	0.011640480
LECT1	2,073242774	3,52722E-10		LECT1	0.7316018867	0.056214765
MMP9	2,373232832	5,48088E-10		MMP9	0.02715222150	0.945520879
MMEL1	3,599237766	1,87916E-14		MMEL1	0.02666021019	0.945720074
MOXD1	3,25804939	1,3944E-23		MOXD1	0.23970001577	0.4587874404
POSTN	2,832912181	1,06319E-11		POSTN	0.14296033062	0.7780729943
ROR2	2,225722267	3,63308E-18		ROR2	0.50190385756	0.1187631094
SIT1	2,528683236	4,56323E-09		SIT1	0.85398179670	0.0019311724
SLC34A2	3,015907839	3,09943E-13		SLC34A2	0.04322631485	0.9107578019
TSPAN19	2,518618103	1,78963E-06		TSPAN19	2.51861810275	0.0001599702
ULBP1	2,213250722	3,9782E-11		ULBP1	0.36480094343	0.2952411726
XG	2,206443316	2,10978E-05		XG	0.37998125594	0.3303205836
**Angiogenesis associated genes**						
**Down-regulated in TMEM230 high vs TMEM230 low**						
**Gene name**	**log2FC**	**padj**		**Gene name**	**log2FC**	**padj**
TMC3	- 2,503399205	4,71168E-08		TMC3	0.53180330413	0.0620561377

Up-regulated in TMEM230 high vs TMEM230 low						
**Gene name**	**log2FC**	**padj**		**Gene name**	**log2FC**	**padj**
HOXA3	4,342084814	4,16713E-05		HOXA3	0.37793180116	0.2205294254
HOXA7	7,695907211	1,02553E-22		HOXA7	0.52021719543	0.1257013868
HOXB3	4,284650669	2,64264E-24		HOXB3	0.14590526890	0.6926876030
HOXB13	3,743738029	4,55944E-06		HOXB13	0.832649867786	0.1274102550
CXCR3	2,54484793	4,18208E-08		CXCR3	0.412711030037	0.2268469735
COL8A1	2,465517007	4,97546E-16		COL8A1	0.157209866058	0.6836562148

Supplementary Table S7. Glycoproteins and angiogenesis gene expression are elevated in GBM compared ODG. Base mean expression of glycoprotein and angiogenesis associated genes in high-grade oligodendroglioma and GBM with high expression of TMEM230 with P value < or = to 0.05.

ODG		GBM	
**Glycoproteins**			
**Gene name**	**baseMean**	**Gene name**	**baseMean**
ABCC3	45.0683380176947	ABCC3	1444.03706547031
ANO9	0.735428106141748	ANO9	2.06291727570392
CD2	9.70675142915889	CD2	54.6693306121704
CD3D	3.31277664846862	CD3D	22.6200940544139
CELSR1	49.2732791098697	CELSR1	310.201.181.467.494
COL2A1	44.0569691986772	COL2A1	1108.10448213783
CXCR6	3.95329276712741	CXCR6	22.0912783463787
C6	2.33767090315604	C6	7.46114605159741
CHI3L1	2215.66437763646	CHI3L1	94488.2736314881
CHI3L2	353.13343598619	CHI3L2	7663.53239764535
EMILIN3	192.344655599255	EMILIN3	1018.87407884725
EVC2	16.2718066760163	EVC2	99.102.443.919.267
GPR1	2.51686109940378	GPR1	43.6084139941885
GPRC5A	16.742289004299	GPRC5A	136.543850100943
GZMA	6.95484889286462	GZMA	38.3824819388928
IGFBP2	951.71535409566	IGFBP2	11055.1760938473
KLRB1	2.59473692383721	KLRB1	11.5174512260351
KISS1R	2.92735554773466	KISS1R	26.7114854318749
LECT1	11.7334609101467	LECT1	43.2152881751178
MMP9	41.4173790666884	MMP9	1082.94127099192
MMEL1	7.1979931723461	MMEL1	29.096.665.053.022
MOXD1	198.507960260697	MOXD1	1689.29149295905
POSTN	31.108628167365	POSTN	4883.03932459562
ROR2	17.5302156073053	ROR2	63.6565646911659
SIT1	1.74217057465497	SIT1	11.5372923448428
SLC34A2	6.150389492759	SLC34A2	61.539.409.311.072
TSPAN19	1.07070368104308	TSPAN19	1.48204534564531
ULBP1	6.01723862239876	ULBP1	38.238313892902
XG	1.59773272868553	XG	6.14828199501641
**Angiogenesis genes**			
**Gene name**	**baseMean**	**Gene name**	**baseMean**
HOXA3	8.56032761824698	HOXA3	115.686704979465
HOXA7	27.0949336848246	HOXA7	343.631701587873
HOXB3	16.7324138948787	HOXB3	302.743789089478
HOXB13	5.24727219896883	HOXB13	83.7034211445756
CXCR3	2.14863192983004	CXCR3	11.381496231888
COL8A1	26.60804657927	COL8A1	353.645518719561

Supplementary Table S8. Genes and pathways identified with increased glioma grade and TMEM230 upregulation in patients with astrocytoma. TMEM230 regulates components of endomembrane system associated with cargo trafficking and secretion: Golgi complex and endoplasmic reticulum dependent

as identified in ODG. Expression analysis correlated all genes differentially expressed with up-regulation of TMEM230 (p adjusted value of < or = 0.05, Supplementary Table 1). Gene Ontology and biological pathways were assessed using the False Discovery Rate.

<table>
<tr><th colspan="2">Endomembrane system genes modulated in ASTROCYTOMA
with TMEM230-high vs TMEM230-low</th></tr>
<tr><th colspan="2">Glycosylation site:N-linked (p-adjusted value 1.35531E-64)</th></tr>
<tr><th>Genes Down-regulated</th><th>Genes Up-regulated</th></tr>
<tr><td>NDST4, IGLON5, UNC5A, CACNA2D3, CACNA2D2, ADAMTS19, PRRT2, BTBD17, CADM2, GRIN3A, STRC, WFIKKN1, KCNC1, GPR21, TMEFF2, CHST1, DSCAML1, GPR45, ADAM29, GLRA3, CSMD3, CSMD2, CSMD1, WNT7B, GLRA4, LGR5, UPK2, GPR61, KCNH3, SLC9A2, OLFM1, SLC9A5, IGFALS, NPTXR, GRIA2, NPTX1, GRIA3, GRIA4, G6PC2, LRIT2, SPRN, KCNK7, ELFN2, OVGP1, GRID2, SEZ6L, ADAMTS20, IL1RAPL1, LRTM2, NTN4, SLC5A12, SLITRK5, LINGO1, PCDH7, MDGA2, KCNK3, KCNK4, SPON1, ICAM5, BSN, EPHB1, SCN1A, ISLR2, EPHA10, PCDH15, MST1R, SCN3B, SCN3A, NTNG2, PCDH20, LRRN4, ST8SIA3, SCN2A, PTPRT, PTPRR, GRIK2, KCNT1, CRTAC1, MME, SLC32A1, MST1, SYP, SORCS3, SYN1, GABBR1, GRIN1, CELSR3, ADAM11, LMTK3, SCN8A, SLC1A1, CACNA1B, CACNA1A, SLC1A4, SLC1A6, CACNA1H, FUT3, FUT6, FUT5, KCNV2, FUT9, SYT2, FGF17, BMP2, CNTN3, GRM4, GALNT9, SSTR1, SSTR2, SSTR5, VCAN, CDHR1, CDHR5, INA, SHANK2, PRTG, SLIT1, CHGA, ACVR2B, FAM155A, COL7A1, GPR173, MYC, GPR179, CHGB, BCAN, GPLD1, PHYHIPL, FAM171A2, GALR3, FLRT1, PCDHA5, PROM2, WNT10B, SSPO, GALNTL6, GABRB3, MRC1, RSPO3, RNF150, SLC2A13, TET1, TMEM179, ANGPTL2, RGR, RNF165, RPRM, AOC2, GABRA5, GABRA3, PRSS35, SULF2, SMOC1, CHRM1, CHRM4, EPX, SLC8A2, CDH7, FGF9, TNR, SEMA6B, SLC15A2, RTN4R, SLC26A1, KIAA1644, DRD2, DRD5, CSPG5, SERINC5, AMH, SGCG, RTN4RL2, VSTM2A, NLGN2, LUZP2, MGAT5B, CACNG2, ANGPT4, SEMA4A, SEMA4B, SEMA4G, GLRB, CBLN2, CBLN1, VIPR1, VIPR2, EYS, CRHR1, SNAP91, IZUMO1, NOG, GFRA1, GP1BA, CCKBR, MAPT, GABRD, CA11, LYVE1, LY6G5B, GALNT13, FAIM2, TMEM145, CHRNB2, DSCAM, PRSS55, SHISA7, SCG3, CHRNA4, LPL, HRH3, CDH20, CDH22, KLRC3, PTCH1, LRP1B, EFNA3, EFNA2, NMUR2, GLP1R, MEGF11, HAPLN1, LRRTM3, LRRTM4, LRRTM1, LRRTM2, TAS2R5, TAS2R4, PCDHGA6, ABCC8, PCDHGA3, OR2H2, PCDHGA1, CDH10, CDH13, CDH18, HCN3, NRXN1, NRXN2, DLL1, LRIG1, OPCML, NEGR1</td><td>ADAMDEC1, TFRC, F11R, IL4I1, ISLR, LIPC, C3AR1, VSTM1, RNASE6, RNASE3, RNASE2, HSPG2, SPINT1, AGTR1, PLBD1, HS3ST3A1, ABCB4, FPR1, IL20RA, CLCNKB, PDGFA, FPR3, FPR2, C2, C3, ADAMTS14, C6, C7, KCNMB1, C19ORF38, NGFR, HLA-DRB5, ABCA5, FUCA1, APCDD1L, FUCA2, FN1, CP, COL1A1, IL22RA1, COL1A2, DCHS2, NOX4, GPR18, HLA-DRB1, PIGT, SIGLEC9, HEXB, LRRC32, OLR1, CTLA4, CTBS, HLA-DPA1, IKBIP, GPR39, CHST6, GPR37, BGN, EMP2, EMP3, F3, SUMF1, PIGB, GPRC5A, SLC7A7, TLR10, CHST2, CRB2, LRRC15, PTAFR, CLECL1, ABCB11, FSTL1, EGFR, SLC7A3, PTGS1, HLA-DMA, HLA-DMB, FRZB, GPA33, TMEM67, SPP1, C11ORF45, IL12RB1, LRRC25, EVC2, P2RY10, GPR55, ADAM33, VWA3A, PLXDC2, DCBLD2, LRG1, SLCO3A1, HLA-DPB1, SIGLEC1, LGR6, SIGLEC7, SIGLEC5, OLFML2B, GPR65, HP, LY75, F13A1, PLOD3, IL1RAP, EFEMP2, EFEMP1, IBSP, ADORA3, ADORA1, HLA-DOA, HLA-DOB, TNFRSF4, TICAM2, TPST1, NAAA, ROR1, ROR2, PLA2R1, DSC2, CHODL, CD274, KCNE1, KCNE3, KCNE4, TACSTD2, LY96, TNFRSF11B, GPR84, GPR82, TMEM26, TNFRSF14, CPA4, CD276, TFPI2, B3GALT4, LY86, LRRC66, TMEM17, PTPRC, OGN, HLA-DRA, KCNK5, ITGB1, MOXD1, ST6GALNAC2, ITGB4, ITGB3, ITGB2, LECT1, FASLG, ECE1, ITGAL, WISP1, TNFSF13B, SPN, FCGR3A, FCGR3B, ITGB8, ITGB7, TNFSF12-TNFSF13, PTCRA, ITGA4, ITGA3, RNASET2, ITGA2, TNFRSF19, ITGA1, TNFRSF1A, NPC2, ITGA7, DSG2, ITGA5, ACPP, COL15A1, C1S, C1R, IL2RG, TSPAN31, CECR1, CCL2, FCGR1A, LRRC8E, CTHRC1, SLC12A3, GZMA, NAGA, GZMH, FCGR2A, IL2RA, IL2RB, PDCD1, FCGR2B, FCGR2C, PRF1, LDLRAD2, ICAM4, IFI30, LCTL, ICAM1, C4A, PTGFRN, EPHB2, SLC12A7, CER1, SLC34A2, C9ORF135, ENTPD2, MUSK, KCNK13, VWA5B1, FNDC7, SFRP4, FNDC1, MXRA8, CD226, EPHA1, MXRA5, CES1, LPAR1, CD1D, PCDH18, CD19, ARSJ, SLAMF8, ST8SIA4, ARSI, SLAMF7, SLAMF6, ARSF, CD14, A2M, APOB, ARSD, ARSE, SLAMF1, SLC10A4, CCR1, CTSA, MSR1, SLC10A6, TNFSF14, TNFSF13, PILRA, BST2, BST1, LPAR6, CALU, CD28, SDC1, CD27, NRP1, FKBP10, CD40, ECM2, CTSZ, SECTM1, GRIK1, CD3G, CTSW, CD3D, PCSK5, EDA2R, CTSS, CTSK, CD37, CCR7, CCR4, CD33, CTSC, CCR2, CTSB, CD53, IL10, CD52, IL15, ANO9, ANO6, MMEL1, OSMR, IL1A, CD48, CD44, CD63, RAMP3, SLC43A3, GBA, OS9, GPC3, CD58, MICB, CD74, CD72, YIPF1, DKKL1, GLB1L, CD69, CD68, CD86, CD80, MPL, LOXL3, LOXL4, PLAT, CELSR1, PLB1, LOXL2, FAM198A, PLAU, FAM198B, CCRL2, FAM3B, ENPP1, FAM3D, PAMR1, CD96, SCARA3, CD93, SPINK8, CYBB, PCOLCE, MR1, PRLR, PDGFRL, OSCAR, ADAM19, SCNN1G, PROCR, CD8B, SCNN1B, ADAM12, DPEP2, HHIPL2, DPEP1, PLD5, KIR2DL4, VCAM1, CRTAM, RARRES1, LAYN, GGH, IL6, PROC, IL7, COL5A2, MANSC1, IL7R, LGALS3BP, TNFAIP6, LAMC3, CPXM2, SIRPB2, LAMC1, CA9, A4GALT, HS3ST3B1, MMP2, MMP9, MMRN1, TMEM217, CHI3L2, CHI3L1, LAMA2, PKD1L1, KDELC2, MAN1C1, TMEM209, OR51E1, GALNT5, GALNT4, GALNT3, EGF, TPBG, LAMB4, LAMB1, SOD3, GALC, CD2, CD4, CD6, CD5, CD7, PTX3, CPVL, GLA, SERPINE1, COL12A1, MRGPRF, GLDN, FGFRL1, GPR171, PRIMA1, CUBN, SERPINF1, SERPINF2, DKK1, GPR160, SERPING1, CFB, ENPEP, CFH, CFI, GNS, SERPINH1, PROM1, ICOS, HLA-DQA2, HLA-DQA1, FZD1, ST14, FZD3, FZD2, ACE, MCAM, FZD7, FZD6, PCDHB1, TNFSF8, SERPINA3, SERPINA1, PRSS23, PHEX, ASGR2, SERPINA5, ADAMTSL1, MRC2, GPR132, C1QTNF1, ADAMTSL4, TCTN2, TCTN1, EMB, TIMP1, HAVCR2, SERPINB2, SLC2A10, FCRL6, NFAM1, IFNGR2, BACE2, CD200R1, ANGPTL7, ANGPTL6, COL21A1, ANGPTL4, HLA-DQB1, ENG, NPSR1, GRN, GCNT1, AFP, GPNMB, OLFML3, OLFML1, AGA, APOL1, GPR156, RNF130, IL10RA, GPR1, SULF1, GPR141, TMEM158, TMEM159, CLCF1, PLP2, FMOD, CCL28, CSF3R, PROS1, TNC, CLU, CDH6, CDH4, PODNL1, LAMP3, CLEC5A, SLC39A8, IL13RA2, IL13RA1, IGFBP4, CD180, FKBP9, RHCG, SLC26A2, CD151, AEBP1, HMMR, CLEC7A, BTLA, IGFBP7, SLC15A3, CD300LF, PLTP, STRA6, CD164, SLC37A2, CD163, BTN2A2, CYBRD1, PXDNL, C1ORF101, P4HA2, ALK, CPM, COL14A1, ADRA1D, TREM2, CPZ, TREM1, SCPEP1, ADAMTS3, ADAMTS1, EMILIN3, EMILIN2, EMILIN1, LBP, GAL3ST4, TRPC7, SLC13A3, CR1, VWF, ASPN, TNMD, PECAM1, FRRS1, UNC93B1, SEMA3A, FGL2, SEMA3F, P2RY8, P2RY6, TTR, P2RY2, P2RY1, IL21R, CEACAM21, TIGIT, MEST, ANGPT2, ANGPT1, MFAP5, MFAP4, NFASC, CLEC2B, P2RX1, PI15, EVI2B, FOLR1, CBLN3, UMODL1, AQP4, KLHDC7A, ANTXR2, AQP1, NIPAL2, SIL1, ICOSLG, HABP2, HLA-H, SLC6A16, IL1R1, SLC11A1, IL1R2, HLA-B, HLA-C, HLA-A, HLA-F, HLA-G, GBGT1, HLA-E, TWSG1, CD109, HMSD, CD101, SLC22A4, GYPC, TMTC2, CD79B, OTOA, CDSN, LIF, GDF5, VASN, VNN1, VNN2, COL18A1, TRAM1, SLC44A3, RYR3, ERMAP, BTN3A1, INSRR, BTN3A3, PDCD1LG2, BTN3A2, IL17RC, NAALADL2, NAALADL1, TLR1, CEACAM1, B3GNT8, CDCP1, B3GNT7, B3GNT5, CEACAM4, TLR8, VWA1, FAM20C, TLR7, TLR6, FAM20A, CD302, TLR5, TLR3, MATN2, TLR2, LILRA6, PTGER4, C1QA, TNXB, PTGER1, PTGER2, AMIGO2, GDPD2, CXCR4, CXCR6, LILRA1, CST7, LILRA2, LILRA4, LILRA5, EGFLAM, CXCR3, CXCR2, SUSD3, LAIR1, MPZL2, GDF15, SUSD2, LILRB1, LILRB2, FAP, LRRN4CL, FAS, F2RL2, FCGBP, STEAP3, STEAP4, HFE, SIRPG, CLEC18C, CLEC18B, TMEM140, CLEC18A, SIT1, C1RL, SVEP1, POSTN, CD300A, EPDR1, COL4A2, LOX, COL4A1, APLNR, RELL1, CD300C, FBLN7, CHRNA1, KLRB1, CHRNA6, CHRNA9, FBLN1, CSF2RB, LTBP2, LTBP1, FBLN5, SCUBE2, DPP4, EFNB2, HRH1, MAN2A1, KLRC1, TGFB2, TGFB3, EPHX3, SLC2A9, NMUR1, KLRD1, ULBP2, FGFR1, ULBP3, ABCD2, MEGF10, PCDHGB3, SLC40A1, TMEM107, BDKRB2, BDKRB1, GUSB, ABCC3, PTGIR, PLAUR, WNT16, TGFBR1, DCN, TGFBR2, TMEM2, IGDCC4, COL6A2, COL6A3, LTB, PAPPA2, CDH19, LTF, COLEC12, CLEC12A, THBS2, THBS4, ACAN, MUC1, ERBB2, S1PR3, S1PR4, LGI4, PMP22, LTBR</td></tr>
<tr><td colspan="2"></td></tr>
<tr><th colspan="2">Endoplasmic reticulum Lumen (p-adjusted value 5.19722 -04)</th></tr>
</table>

Genes Down-regulated	Genes Up-regulated
SPON1, WNT7B, COL7A1	FKBP10, COL18A1, SERPINA1, COL14A1, COL12A1, CTSZ, ADAMTSL1, LIPC, ADAMTSL4, B2M, SIL1, CTSC, RNASET2, GPX8, PLAUR, PDIA5, PDIA4, SUMF1, COL4A2, COL4A1, COL6A2, COL8A2, COL6A3, COL8A1, COL21A1, RDH5, ERP27, CES1, COL15A1, MTTP, PDGFA, OS9, KDELC2, ARSJ, SERPINH1, ARSI, ARSF, ARSD, APOB, ARSE, COL1A1, COL3A1, CD4, PROC, COL1A2, COL5A1, P4HA2, COL5A2
**Endocytic vesicle membrane (p-adjusted value 1.729E-02)**	
**Genes Down-regulated**	**Genes Up-regulated**
GRIA2, WNT7B, PTCH1, DLG4, CACNG2, GRIA3, GRIA4	OLEC12, MSR1, CD74, CD163, HLA-DRB5, CAV1, HLA-DPB1, HLA-DRA, HLA-DQA2, HLA-DQA1, HLA-DQB2, HLA-DRB1, HLA-DPA1, HLA-DQB1
**Proteoglycans in cancer (p-adjusted value 2.85869-04)**	
**Genes Down-regulated**	**Genes Up-regulated**
MYC, PRKCG, PRKCB, PIK3R1, WNT10B, WNT7B, PTCH1	ITGB1, ITGB3, PIK3CD, FASLG, PIK3CG, PLAU, PLCE1, HGF, MMP2, ITGA2, PLAUR, WNT16, HSPG2, MMP9, DCN, HCLS1, ITGA5, CD44, TLR2, CD63, SDC4, TWIST1, IQGAP1, HOXD10, EGFR, PIK3R5, RRAS, ERBB2, GPC3, PLCG2, FLNA, FLNC, FZD1, TGFB2, FZD3, FZD2, CAV2, LUM, FZD7, CAV1, FZD6, IGF2, FN1, MSN, FAS, SDC1, PTPN6, FGFR1
**Proteoglycan (p-adjusted value 1.766833 E-03)**	
**Genes Down-regulated**	**Genes UP-regulated**
BCAN, VCAN, TNR, CSPG5	CD74, NRP1, SDC4, LUM, BGN, CXCR4, HSPG2, DCN, ACAN, SRPX2, GPC3, SDC1, FMOD, CD44

Supplementary Table S9. Genes and pathways identified with increased glioma grade and TMEM230 upregulation in patients with astrocytoma. TMEM230 regulates motor protein dependent intracellular trafficking and secretion of metalloproteinases (ADAMs and MMPs) and phagosomes for microchannel formation as identified in ODG. Expression analysis correlated all genes differentially expressed with up-regulation of TMEM230 (p adjusted value of < or = 0.05, Supplementary Table 1). Gene Ontology and biological pathways were assessed using the False Discovery Rate method.

**Trafficking and secretion associated genes modulated in ASTROCYTOMA with TMEM230-high vs TMEM230-low**	
**Secreted (p-adjusted value 5.30206E-27)**	
**Genes Down-regulated**	**Genes Up-regulated**
IGLON5, FAM19A1, CHGB, BCAN, GPLD1, ADAMTS19, FLRT1, WNT10B, BTBD17, SSPO, WFIKKN1, C1QTNF4, RSPO3, TMEFF2, ANGPTL2, WNT7B, PRSS37, PRSS35, SMOC1, FGF9, TNR, OLFM1, IGFALS, AMH, C1QL4, ADAMTS20, CRH, VSTM2A, LUZP2, NTN4, THSD4, TUB, ANGPT4, SST, CBLN2, CBLN1, SPON1, EYS, STX1A, EPHA10, PCDH15, NMB, CRTAC1, NOG, MST1, DNASE1L2, NPW, CGREF1, CA11, GABBR1, LY6G5B, DSCAM, SCG3, MASP2, LPL, TAC1, FGF17, BMP2, HAPLN1, CORT, VCAN, SLIT1, CHGA, COL7A1	ADAMDEC1, TFRC, COL12A1, SERPINE1, GLDN, ISLR, LIPC, NAMPT, VSTM1, SERPINF1, SERPINF2, RNASE6, RNASE4, RNASE3, HSPG2, DKK1, SPINT1, SERPING1, CFB, CFD, CFH, DHH, CFI, PDGFA, PLA2G5, C2, C3, ADAMTS14, C6, C7, SCGB1D2, S100A13, SFN, ICOS, GSDMD, ACE, FUCA2, FN1, LYZ, CP, COL1A1, COL1A2, HAMP, SERPINA3, SERPINA1, ELN, ADM, PRSS23, SERPINA5, ADAMTSL1, C1QTNF2, MMP25, C1QTNF1, ADAMTSL4, GNLY, TCTN1, OLR1, TIMP1, ANXA1, FGFBP2, SERPINB2, ANXA2, BGN, F3, MMP11, CD200R1, ANGPTL7, ANGPTL6, COL21A1, ANGPTL4, CRB2, GRN, CTF1, AFP, FSTL1, EGFR, APOL4, SRPX2, CNPY4, FRZB, OLFML3, OLFML1, SPP1, APOL1, C11ORF45, GSN, RNLS, CCL20, VWA3A, COL3A1, LRG1, CLCF1, C16ORF89, SIGLEC1, FMOD, CCL28, OLFML2B, CSF3R, PROS1, HP, TNC, F13A1, IL1RAP, FGF1, OLFML2A, CLU, CXCL14, EFEMP2, PODNL1, EFEMP1, IBSP, IGFBP5, IGFBP4, IGFBP2, THNSL2, PLA2R1, S100A9, S100A8, LY96, C1ORF54, TNFRSF11B, AEBP1, IGFBP7, IGFBP6, PLTP, CPA5, CPA4, CD164, CD163, TFPI2, LY86, PXDNL, CXCL10, CXCL11, OGN, RLN2, ANXA2P2, COL14A1, LECT1, FASLG, TREM2, TRH, CPZ, TREM1, WISP1, TNFSF13B, SCPEP1, FCGR3A, FCGR3B, ADAMTS3, MDK, ADAMTS1, EMILIN3, EMILIN2, EMILIN1, TNFSF12-TNFSF13, LBP, VWF, RNASET2, ASPN, TNFRSF1A, NPC2, XCL2, XCL1, ANG, ACPP, COL15A1, SEMA3A, FGL2, TULP2, SEMA3F, CECR1, TTR, CCL5, CCL2, CTHRC1, ANGPT2, ANGPT1, GZMA, MSMP, APLN, MFAP5, GZMK, MFAP4, APOC2, APOC1, PI15, FOLR1, CBLN3, SPON2, PRF1, ICAM4, IFI30, ANTXR2, C4A, LGALS3, LGALS1, LGALS9, B2M, CER1, HABP2, IL1R1, IL1R2, HLA-C, VWA5B1, FNDC7, CHIT1, SFRP4, TWSG1, SLPI, FNDC1, CRISPLD1, HMSD, COL8A2, COL8A1, MXRA5, SDC4, OTOA, ARSJ, ARSI, ARSF, CD14, A2M, APOB, SLAMF1, CDSN, TNFSF14, TNFSF13, IGF2, LIF, PILRA, GDF5, VASN, CALU, SDC1, NRP1, COL18A1, CD40, ECM2, SECTM1, PCSK5, ESM1, CTSB, IL10, IL15, IL18, IL16, MMEL1, PDCD1LG2, OTOS, IL1A, CEACAM1, CDCP1, TFF3, VWA1, FAM20C, FAM20A, MATN2, C1QB, C1QA, CD63, TNXB, CAPG, CST7, LILRA2, LILRA5, CST3, EGFLAM, GPC3, IL32, GDF15, DKKL1, LILRB1, CD40LG, FAP, GLB1L, SAA1, SAA2, FAS, C1QC, FCGBP, CLEC18C, LOXL3, LOXL4, CLEC18B, PLAT, CLEC18A, LOXL1, LOXL2, FAM198A, PLAU, C1RL, FAM3B, ENPP1, FAM3D, SVEP1, FAM150B, PAMR1, POSTN, SPINK8, EPDR1, PCOLCE, NPNT, MR1, PRLR, PDGFRL, OSCAR, COL4A2, CD8B, LOX, COL4A1, CD8A, ADAM12, HHIPL2, FBLN7, HTRA3, FBLN1,

	LTBP2, NTS, LTBP1, FBLN5, SCUBE2, DPP4, C6ORF15, CHAD, FGF20, CYTL1, TGFB2, TGFB3, RARRES2, GGH, EPHX3, ISG15, SSC5D, IL6, PROC, COL5A1, IL7, COL5A2, TGFBI, ULBP2, IL7R, LGALS3BP, CXCL9, C2ORF40, LAMC3, CPXM2, LAMC1, CASP4, MMP7, MMP2, PLAUR, WNT16, MMP9, DCN, MMRN1, OAS1, COL6A2, CHI3L2, CHI3L1, COL6A3, PAPPA2, LTF, LAMA2, C2ORF66, THBS4, ACAN, MUC1, GBP1, LGI4, LUM, PLA2G2A, LAMB4, LAMB1, SOD3, CD6, C9ORF47, PTX3
**Phagosome (p-adjusted value 8.113E-09)**	
**Genes Down-regulated**	**Genes Up-regulated**
MRC1, ATP6V1G2, TUBA8	TGB1, NCF1, TFRC, NCF2, ITGB3, NCF4, ITGB2, TCIRG1, CTSS, MRC2, TUBA1C, TUBB6, FCGR3A, FCGR3B, SEC61G, OLR1, HLA-DOA, HLA-DOB, HLA-DPA1, ITGA2, HLA-B, HLA-C, TAP1, CYBA, HLA-A, HLA-F, HLA-G, HLA-E, TLR6, ITGA5, PLA2R1, HLA-DQB1, TLR2, COLEC12, C1R, THBS2, THBS4, C3, HLA-DMA, HLA-DMB, CLEC7A, CD14, FCGR1A, HLA-DQA2, HLA-DQA1, MSR1, HLA-DRB5, FCGR2A, HLA-DPB1, HLA-DRA, FCGR2B, FCGR2C, HLA-DRB1
**Motor protein (p-adjusted value 1.240764-03)**	
**Genes Down-regulated**	**Genes Up-regulated**
KIF21B, KIF3C, MYO5A, DNM1, MYO16, DNM3, KIF26A	DNAH2, ACAA2, DNAH5, DNAH6, KIF14, DNAH9, MYL12A, DNALI1, KIF15, DNAI1, DNAH12, DNAH11, DNAI2, DYNLT3, KIF24, KIF23, MYO1E, CENPE, KIF18A, KIF9, KIF16B, KIF4A, MYO5B, MYO5C, DYNLRB2, KIF2C, KIF20A, MYO1

Supplementary Table S10. Genes and pathways identified with increased glioma grade and TMEM230 upregulation in astrocytoma. TMEM230 promoted upregulation of glycoproteins and angiogenesis in highly vascularized infiltrating astrocytoma. Expression analysis correlated all genes differentially expressed with up-regulation of TMEM230 (p adjusted value of < or = 0.05, Supplementary Table 1). Gene Ontology and biological pathways were assessed using the False Discovery Rate method.

**Glycoproteins and angiogenesis associated genes modulated in ASTROCYTOMA with TMEM230-high vs TMEM230-low**	
**Glycoprotein (p-adjusted value 8.74481E-74)**	
**Genes Down-regulated**	**Genes Up-regulated**
NDST4, IGLON5, UNC5A, CACNA2D3, CACNA2D2, ADAMTS19, PRRT2, BTBD17, CADM2, GRIN3A, STRC, WFIKKN1, KCNC1, GPR21, TMEFF2, CHST1, DSCAML1, GPR45, ADAM29,	ADAMDEC1, TFRC, F11R, IL4I1, ISLR, LIPC, C3AR1, VSTM1, RNASE6, RNASE3, RNASE2, HSPG2, SPINT1, AGTR1, PLBD1, HS3ST3A1, ABCB4, FPR1, IL20RA, CLCNKB, PDGFA, FPR3, FPR2, C2, C3, ADAMTS14, C6, C7, KCNMB1, C19ORF38, NGFR, HLA-DRB5, ABCA5, FUCA1, APCDD1L, FUCA2, FN1, CP, COL1A1, IL22RA1, COL1A2, DCHS2, NOX4, FXYD5, GPR18, HLA-DRB1, PIGT, SIGLEC9, HEXB, LRRC32, MMP25, OLR1, CTLA4, CTBS, HLA-DPA1, IKBIP, GPR39, CHST6, GPR37, BGN, EMP2, EMP3, F3, SUMF1, PIGB, GPRC5A, SLC7A7, TMEM106C, TLR10, HCST, CHST2, CRB2, LRRC15, PTAFR, CLECL1, ABCB11, FSTL1, EGFR, SLC7A3, PTGS1, HLA-DMA, SRPX2, HLA-DMB, FRZB, GPA33, TMEM67, SPP1, C11ORF45, IL12RB1, LRRC25, EVC2, P2RY10, SMAGP, GPR55, ADAM33, VWA3A, PLXDC2, DCBLD2, COL3A1, LRG1, SLCO3A1, HLA-DPB1, SIGLEC1, LGR6, SIGLEC7, SIGLEC5, OLFML2B, GPR65, HP, LY75, F13A1, PLOD3, IL1RAP, OLFML2A, EFEMP2, EFEMP1, IBSP, ADORA3, ADORA1, HLA-DOA, HLA-DOB, TNFRSF4, TPST1, NAAA, ROR1, ROR2, PLA2R1, C21ORF62, DSC2, CHODL, CD274, KCNE1, KCNE3, KCNE4, ECSCR, TACSTD2, LY96, TNFRSF11B, GPR84, GPR82, TMEM26, TNFRSF14, CPA4, CD276, TFPI2, B3GALT4, TNFRSF10C, LY86, LRRC66, TMEM17, PTPRC, OGN, HLA-DRA, KCNK5, ITGB1, MOXD1, ST6GALNAC2, ITGB4, ITGB3, ITGB2, LECT1, FASLG, ECE1, ITGAL, WISP1, TNFSF13B, SPN, FCGR3A, FCGR3B, ITGB8, ITGB7, TNFSF12-TNFSF13, PTCRA, ITGA4, ITGA3, RNASET2, ITGA2, TNFRSF19, ITGA1, TNFRSF1A, NPC2, ITGA7, DSG2, ITGA5, ACPP, COL15A1, C1S, C1R, IL2RG, TSPAN31, CECR1, CCL5, CCL2, FCGR1A, LRRC8E, CTHRC1, SLC12A3, GZMA, NAGA, GZMH, FCGR2A, IL2RA, IL2RB, CD248, PDCD1, FCGR2B, FCGR2C, SPON2, PRF1, LDLRAD2, ICAM4, IFI30, LCTL, ICAM1, C4A, PTGFRN, EPHB2, SLC12A7, CER1, SLC34A2, C9ORF135, ENTPD2, MUSK, KCNK13, VWA5B1, FNDC7, SFRP4, FNDC1, MXRA8, CD226, EPHA1, MXRA5, CES1, SDC4, LPAR1, CD1D, PCDH18, CD19, ARSJ, SLAMF8, ST8SIA4, ARSI, SLAMF7, SLAMF6, ARSF, CD14, A2M, APOB, ARSD, ARSE, SLAMF1, SLC10A4, CCR1, CTSA, MSR1, SLC10A6, TNFSF14, TNFSF13, IGF2, PILRA, BST2, BST1, LPAR6, CALU, CD28, SDC1, CD27, NRP1, FKBP10, CD40, ECM2, CTSZ, SECTM1, GRIK1, CD3G, CTSW, CD3D, PCSK5, EDA2R, CTSS, CTSK, RAC2, CD37, CCR7, CCR5, CCR4, CD33, CTSC, CCR2, CTSB, CD53, IL10, CD52, IL15, ANO9, ANO6, MMEL1, OSMR, IL1A, CD48, CD44, CD63, TMPRSS7, RAMP3, SLC43A3, GBA, OS9, GPC3, CD58, MICA, MICB, CD74, CD72, YIPF1, DKKL1, XG, GLB1L, CD69, CD68, CD86, CD80, MPL, LOXL3, LOXL4, PLAT, CELSR1, PLB1, LOXL2, FAM198A, PLAU, MPZ, FAM198B, CCRL2, FAM3B, ENPP1, FAM3D, PAMR1, CD96, SCARA3, CD93, SPINK8, CYBB, PCOLCE, MR1, PRLR, PDGFRL, OSCAR, ADAM19, SCNN1G, PROCR, CD8B, SCNN1B, ADAM12, DPEP2, HHIPL2, DPEP1, PLD5, KIR2DL4, CHAD, C16ORF54, VCAM1, CRTAM, LAYN, GGH, IL6, PROC, IL7, COL5A2, MANSC1, IL7R, LGALS3BP, TNFAIP6, LAMC3, CPXM2,

	SIRPB2, LAMC1, CA9, A4GALT, HS3ST3B1, MMP2, MMP9, MMRN1, TMEM217, CHI3L2, CHI3L1, LAMA2, PKD1L1, KDELC2, MAN1C1, TMEM209, OR51E1, GALNT5, GALNT4, GALNT3, EGF, TPBG, LAMB4, LAMB1, SOD3, GALC, CD2, CD4, CD6, CD5, CD7, PTX3, VIM, CPVL, GLA, SERPINE1, COL12A1, MRGPRF, GLDN, FGFRL1, GPR171, PRIMA1, CUBN, SERPINF1, SERPINF2, DKK1, GPR160, SERPING1, CFB, ENPEP, CFH, CFI, GNS, SERPINH1, PROM1, ICOS, HLA-DQA2, HLA-DQA1, FZD1, ST14, FZD3, FZD2, ACE, MCAM, FZD7, FZD6, PCDHB1, SNAI1, TNFSF8, SERPINA3, SERPINA1, PRSS23, PHEX, ASGR2, SERPINA5, ADAMTSL1, MRC2, GPR132, C1QTNF1, ADAMTSL4, TCTN2, TCTN1, EMB, TIMP1, HAVCR2, SERPINB2, SLC2A10, FCRL6, NFAM1, IFNGR2, BACE2, CD200R1, ANGPTL7, ANGPTL6, COL21A1, ANGPTL4, HLA-DQB2, HLA-DQB1, ENG, NPSR1, GRN, GCNT1, AFP, GPNMB, OLFML3, OLFML1, AGA, APOL1, GPR156, RNF130, IL10RA, GPR1, SULF1, GPR141, TMEM158, TMEM159, CLCF1, C16ORF89, PLP2, FMOD, SLC28A1, CCL28, CSF3R, PROS1, TNC, CLU, CDH6, CDH4, PODNL1, LAMP3, CLEC5A, SLC39A8, GYG2, IL13RA2, IL13RA1, IGFBP5, IGFBP4, IGFBP2, CD180, FKBP9, RHCG, SLC26A2, CD151, AEBP1, HMMR, CLEC7A, BTLA, IGFBP7, IGFBP6, SLC15A3, CD300LF, PLTP, STRA6, CD164, SLC37A2, CD163, BTN2A2, CYBRD1, PXDNL, C1ORF101, P4HA2, ALK, CPM, COL14A1, ADRA1D, TREM2, CPZ, TREM1, SCPEP1, ADAMTS3, ADAMTS1, EMILIN3, EMILIN2, EMILIN1, LBP, GAL3ST4, TRPC7, SLC13A3, CR1, VWF, ASPN, TNMD, PECAM1, FRRS1, UNC93B1, SEMA3A, FGL2, SEMA3F, P2RY8, P2RY6, TTR, P2RY2, P2RY1, IL21R, CEACAM21, TIGIT, MEST, ANGPT2, ANGPT1, MFAP5, MFAP4, NFASC, CLEC2B, APOC2, P2RX1, PI15, EVI2B, FOLR1, CBLN3, UMODL1, AQP4, KLHDC7A, ANTXR2, AQP1, NIPAL2, B2M, SIL1, ICOSLG, HABP2, HLA-H, ACSL1, SLC6A16, IL1R1, SLC11A1, IL1R2, HLA-B, HLA-C, HLA-A, HLA-F, HLA-G, GBGT1, HLA-E, CHIT1, TWSG1, CD109, HMSD, CD101, SLC22A4, GYPC, TMTC2, CD79B, OTOA, TRPM8, CDSN, LIF, GDF5, VASN, VNN1, VNN2, COL18A1, TRAM1, SLC44A3, ESM1, ERMAP, BTN3A1, INSRR, BTN3A3, PDCD1LG2, BTN3A2, IL17RC, NAALADL2, NAALADL1, TLR1, CEACAM1, B3GNT8, CDCP1, B3GNT7, B3GNT5, CEACAM4, TLR8, VWA1, FAM20C, RDH5, TLR7, TLR6, FAM20A, CD302, TLR5, TLR3, MATN2, TLR2, C1QB, LILRA6, PTGER4, C1QA, TNXB, PTGER1, PTGER2, AMIGO2, GDPD2, CXCR4, CXCR6, LILRA1, CST7, LILRA2, LILRA4, LILRA5, CST3, EGFLAM, CXCR3, PDPN, CXCR2, SUSD3, LAIR1, MPZL2, GDF15, SUSD2, LILRB1, LILRB2, CD40LG, FAP, LRRN4CL, FAS, F2RL2, C1QC, FCGBP, STEAP3, STEAP4, HFE, SIRPG, CLEC18C, CLEC18B, TMEM140, CLEC18A, SIT1, C1RL, SVEP1, POSTN, CD300A, HGF, EPDR1, COL4A2, LOX, COL4A1, APLNR, HIST1H2BE, RELL1, CD300C, HIST1H2BC, FBLN7, CHRNA1, KLRB1, CHRNA6, CHRNA9, FBLN1, CSF2RB, LTBP2, LTBP1, FBLN5, SCUBE2, DPP4, EFNB2, HRH1, MAN2A1, KLRC1, TGFB2, TGFB3, CLEC17A, EPHX3, SSC5D, SLC2A9, KIF18A, TMEM119, NMUR1, KLRD1, ULBP2, FGFR1, ULBP3, ABCD2, MEGF10, PCDHGB3, SLC40A1, TMEM107, BDKRB2, BDKRB1, GUSB, ABCC3, PTGIR, PLAUR, WNT16, TGFBR1, DCN, TGFBR2, TMEM2, IGDCC4, OAS2, COL6A2, COL6A3, LTB, PAPPA2, CDH19, LTF, COLEC12, CLEC12A, HIST1H2BJ, HIST1H2BK, THBS2, THBS4, ACAN, MUC1, ERBB2, S1PR3, S1PR4, LGI4, LUM, PMP22, LTBR
**Angiogenesis associated genes (p-adjusted value 1.02218E-05)**	
**Genes Down-regulated**	**Genes Up-regulated**
TMEM100, FGF9, EPHB1, NRXN1, ANGPT4, SEMA4A	OL18A1, NRP1, SERPINE1, TNFAIP2, FGF1, MEOX2, SAT1, PIK3CG, ELK3, ESM1, CYP1B1, TNFSF12-TNFSF13, EPHB2, HOXA7, HTATIP2, VAV3, WARS, TNFRSF12A, ANXA2, SYK, MMP2, IL18, HSPG2, MMP14, CEACAM1, COL4A2, ANGPTL6, COL8A2, PECAM1, HOXB3, COL8A1, ANG, ANGPTL4, ITGA5, EPHA1, COL15A1, ENPEP, SHC1, SH2D2A, ECSCR, PDGFA, PIK3R6, TYMP, SRPX2, CXCR3, CCL2, HMOX1, TGFB2, ANGPT2, ANGPT1, EGF, MCAM, CAV1, FN1, TBX4, MEIS1, FAP, TGFBI, FGFR1

Supplementary Table S11. Genes identified with increased glioma grade and TMEM230 upregulation in hypoxia in ODG and Astrocytoma. Expression analysis correlated all genes differentially expressed with up-regulation of TMEM230 (p adjusted value of < or = 0.05, Supplementary Table 1). Gene Ontology and biological pathways were assessed using the False Discovery Rate method as shown.

**Hypoxia gene modulated in ODG withTMEM230-high vs TMEM230-low**	
**Response to Hypoxia (p-adjusted value 2.702229E-03)**	
**Genes Down-regulated**	**Genes Up-regulated**
CHRNA4, DRD2, CHRNB2, PRKCB, CBFA2T3, BMP2	PLAT, TRH, PLOD1, THBS1, ASCL2, DPP4, PDLIM1, LDHA, PLAU, UCP2, CASP1, PTK2B, HMOX1, CA9, TGFB2, POSTN, MYOCD, TGFB1, VCAM1, ITGA2, AGTRAP, CYBA, ATP1B1, WTIP, SOD2, MT3, SOD3, NR4A2, TGFBR3, ALDH3A1, MMP14, CXCL12, ANG, TLR2

Hypoxia gene modulated in ASTROCYTOMA with TMEM230-high vs TMEM230-low	
**Response to Hypoxia (p-adjusted value 3.42166E-2)**	
**Genes Down-regulated**	**Genes Up-regulated**
CHRNA4, DRD2, CHRNB2, PRKCB, CBFA2T3, BMP2	CXCR4, ADM, PLAT, TRH, ASCL2, LOXL2, DPP4, PDLIM1, CST3, MUC1, PLAU, UCP2, CASP1, ADORA1, CCL2, HMOX1, CA9, TGFB2, POSTN, ANGPT2, VCAM1, TGFB3, MMP2, CAV1, ITGA2, AGTRAP, CYBA, SOD2, SOD3, TGFBR2, ALDH3A1, MMP14, NOX4, ANG, ANGPTL4, ENG, TLR2
